# Supplementary figures and images for: Evidence for Widespread Positive and Negative Selection in Coding and Conserved Noncoding Regions of Capsella grandiflora
Source: PLoS Genet. 2014 Sep 25;10(9):e1004622. doi: 10.1371/journal.pgen.1004622 (PMC4178662; doi:10.1371/journal.pgen.1004622)

Pi

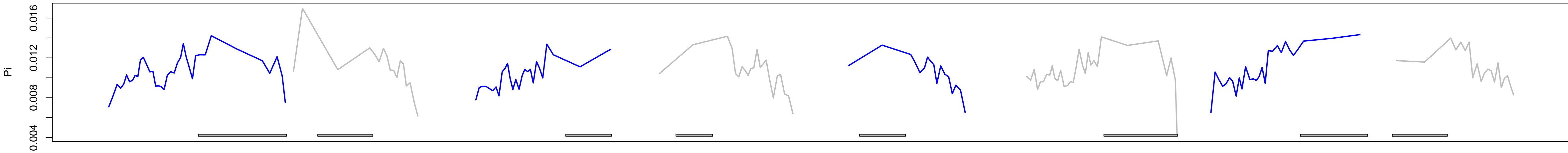

Divergence

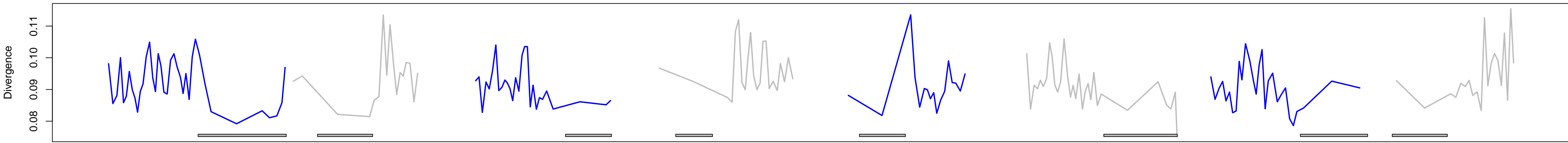

Supplement: Figure S2 — Pairwise diversity and divergence at 4-fold degenerate sites across the entire genome. The x-axis represents position along the genome. Statistics were calculated in windows of 5,000 SNPs. Individual lines alternating between grey and blue represent chromosomes. The location of the centromere on each chromosome is indicated by the grey box along the x-axis. (PDF) [file pgen.1004622.s002.pdf]

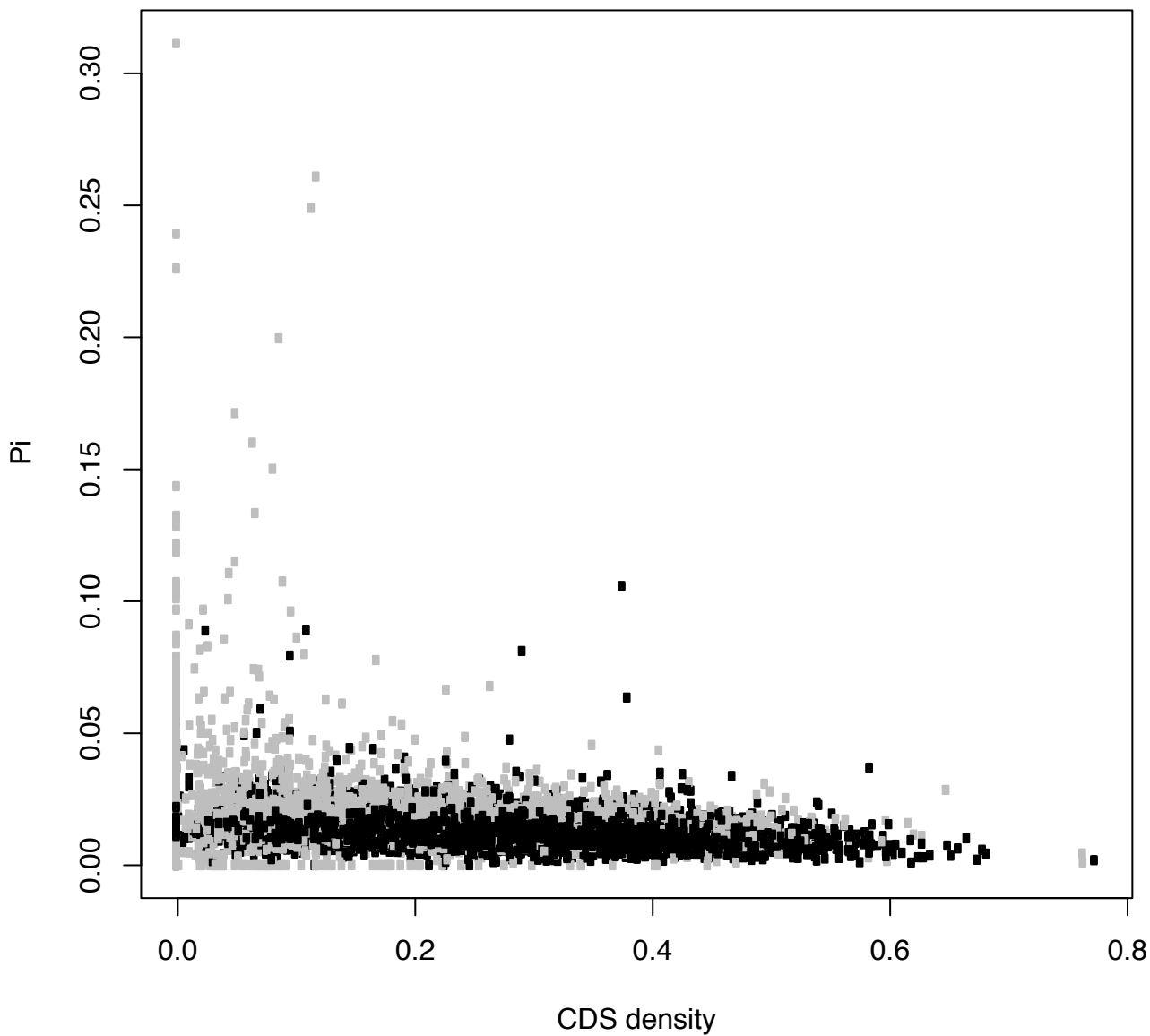

Supplement: Figure S3 — Coding density versus 4-fold degenerate diversity across the genome. Each point represents one 10 kb window. Black points represent windows that do not overlap centromeres while grey points represent windows that do overlap centromeres. There is a slight negative correlation between diversity and coding density both with and without centromeric windows. (PDF) [file pgen.1004622.s003.pdf]

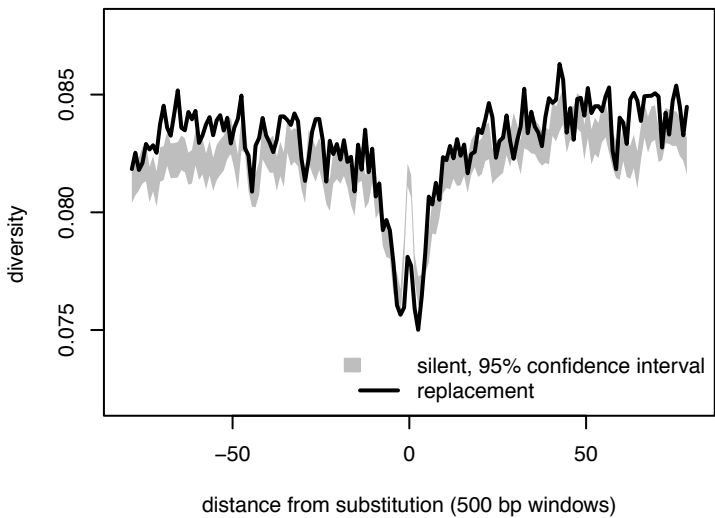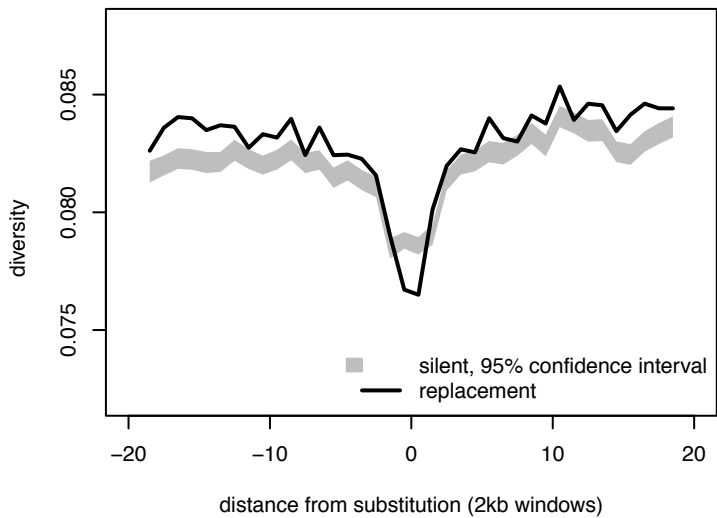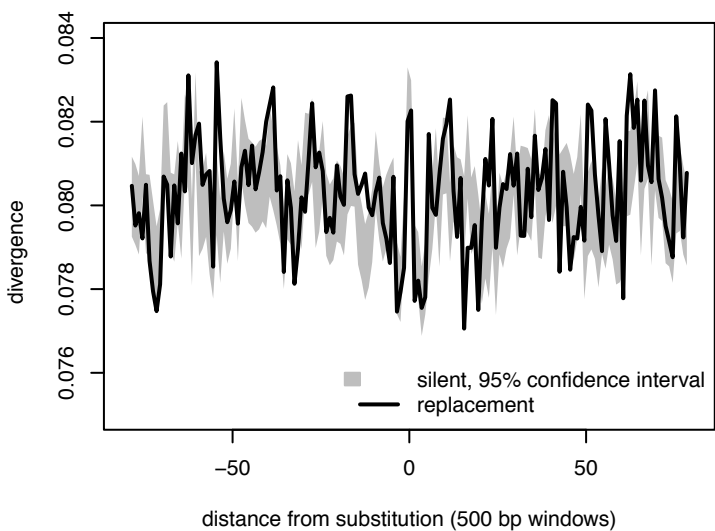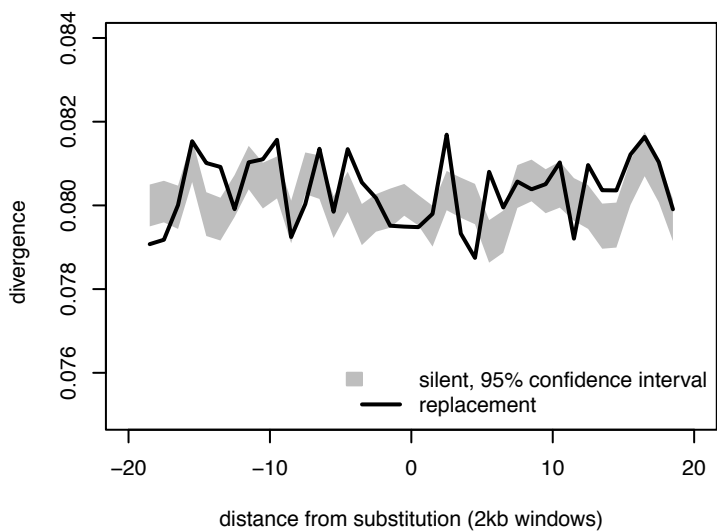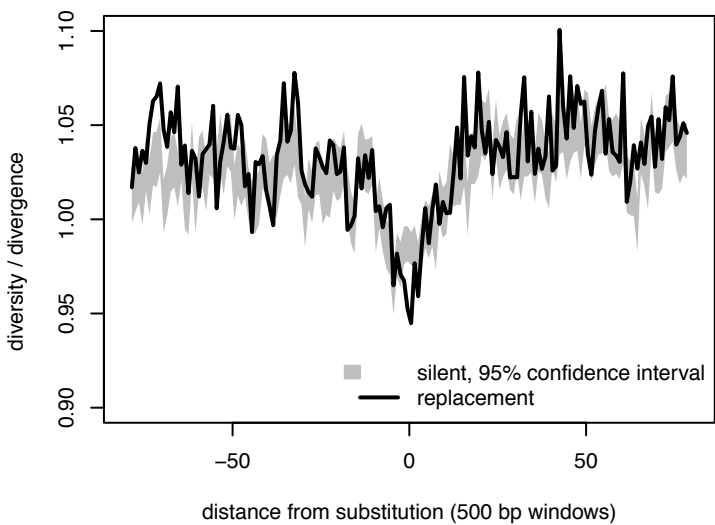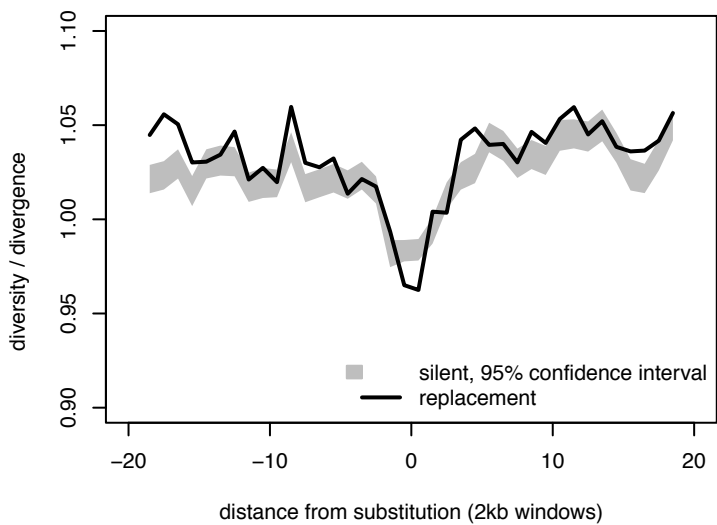

Supplement: Figure S8 — Robustness of sweep analysis to different window sizes. This panel shows the results of our scans for recurrent selective sweeps using alternative window sizes: 500 bp on left and 2 kb on right. Otherwise, the methods are the same as described previously. (PDF) [file pgen.1004622.s008.pdf]

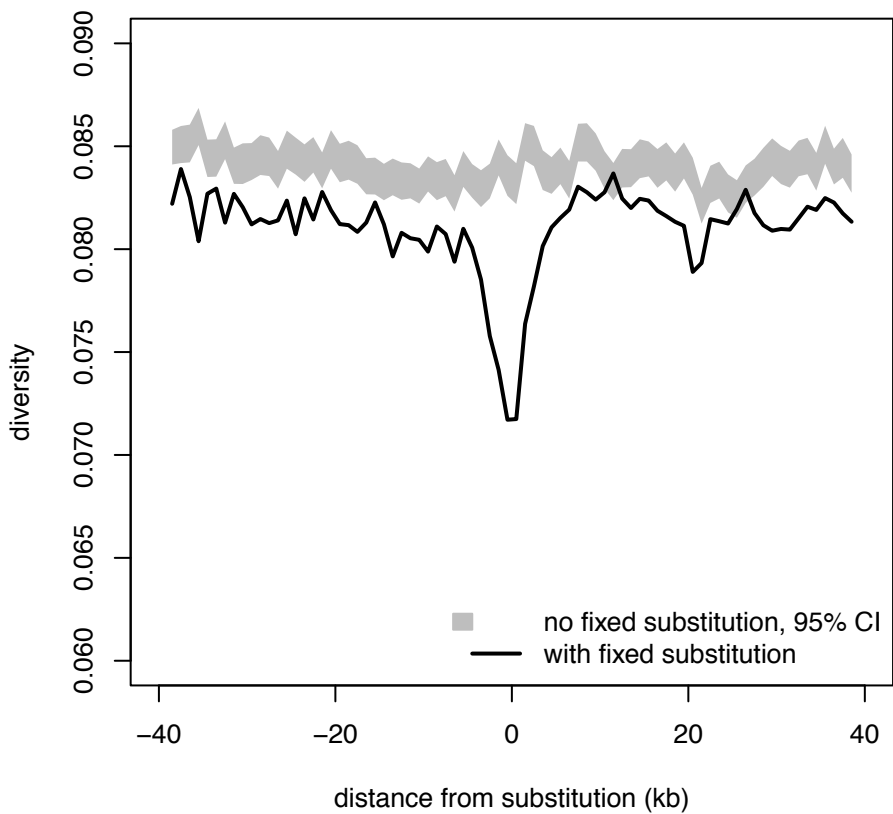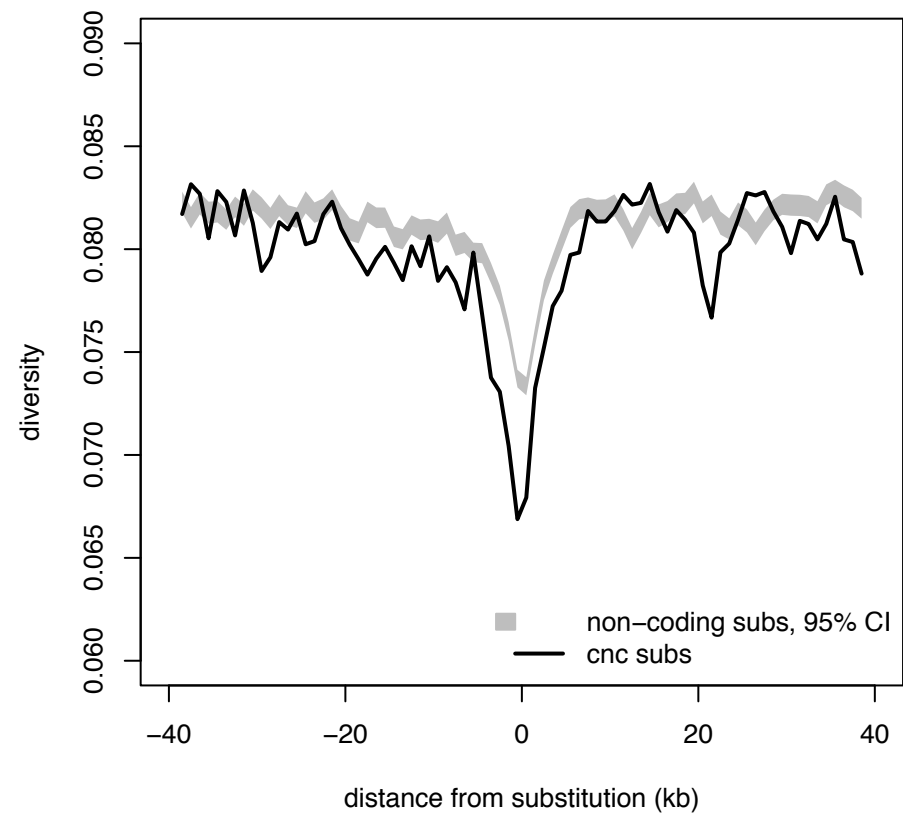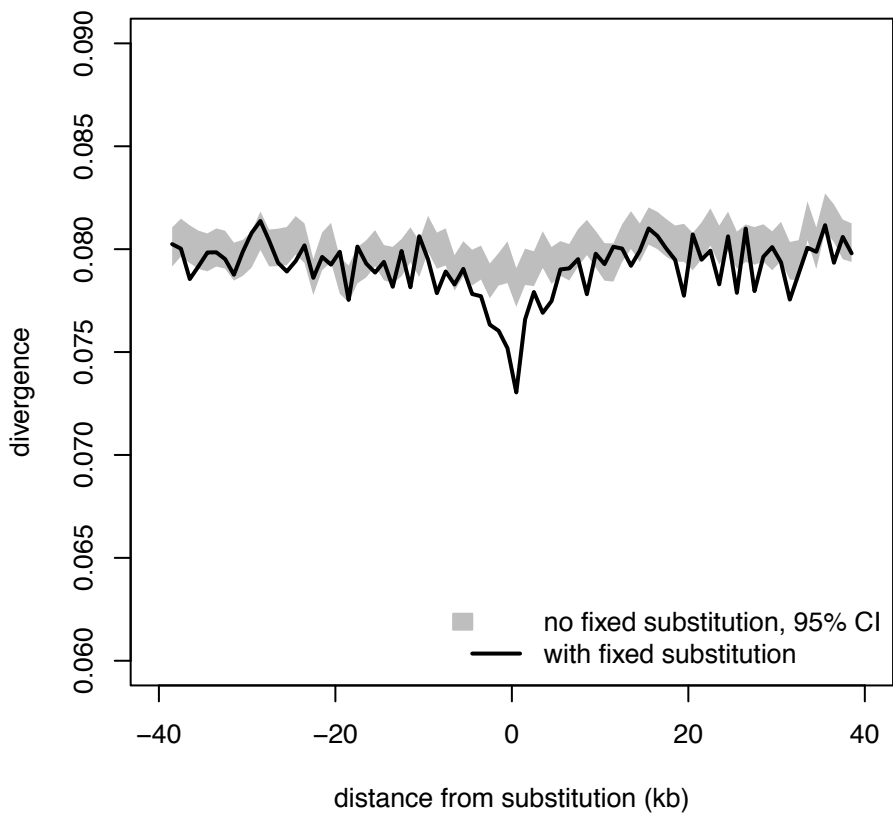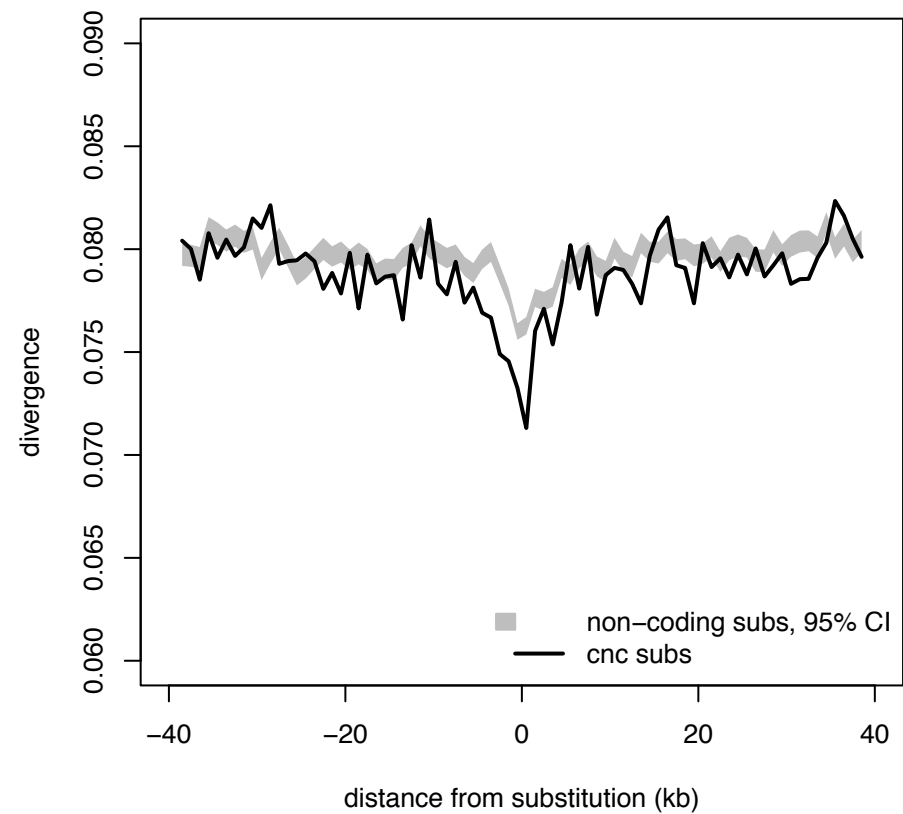

Supplement: Figure S9 — Additional diversity and divergence data for sweeps around substitutions in conserved noncoding regions. The left panels show diversity at 4-fold degenerate sites and divergence at 4-fold degenerate sites around substitutions in conserved non-coding sequence (black lines) and non-conserved intergenic sequence (gray shading represents 95% confidence intervals). The right panels show the same information for diversity and divergence at 4-fold degenerate sites around conserved noncoding sequences containing fixed substitutions (black lines) and conserved noncoding sequences without fixed substitutions (gray shading represents 95% confidence intervals). (PDF) [file pgen.1004622.s009.pdf]

# Site frequency spectra of for replacement substitutions

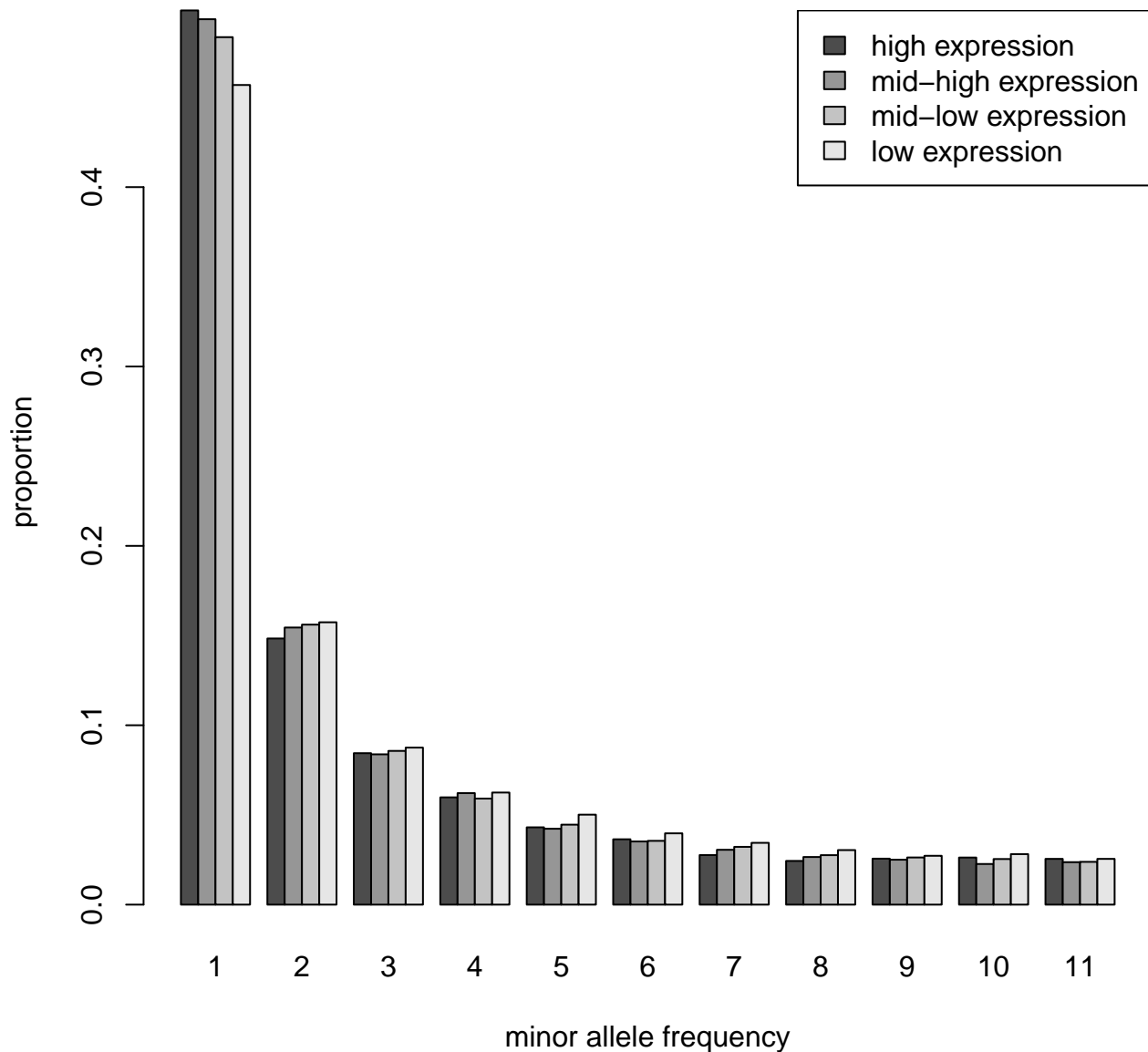

Supplement: Figure S10 — Allele frequency spectra of replacement sites in genes with different expression levels. (PDF) [file pgen.1004622.s010.pdf]
